# Clinicodemographic Profile of Kidney Diseases in a Tertiary Hospital of Central Nepal, Chitwan: A Descriptive Cross-sectional Study

**DOI:** 10.31729/jnma.4972

**Published:** 2020-07-31

**Authors:** Madhav Ghimire, Shreeju Vaidya, Hari Prasad Upadhyay

**Affiliations:** 1 Department of Nephrology, College of Medical Sciences Teaching Hospital, Bharatpur, Nepal; 2 Department of Community Medicine, College of Medical Sciences Teaching Hospital, Bharatpur, Nepal

**Keywords:** *acute kidney injury*, *chronic kidney disease*, *end stage renal disease: sepsis*

## Abstract

**Introduction:**

Spectrum of kidney diseases differs significantly in developing and developed countries. However, there is no central registry regarding the nature of such diseases in Nepal and our center either. The study aims to know the clinicodemographic spectrum of kidney disease patients admitted to our hospital.

**Methods:**

This study was a descriptive cross sectional study done in the department of Nephrology, College of Medical Sciences Teaching Hospital from May 2018 to April 219. Convenient sampling was done and all the consecutive kidney disease patients irrespective of their age, sex, and renal diagnosis were included in the study. Ethical approval was taken from the Institutional Review Committee of the college (reference number. 2016/COMSTH/IRC/049). Clinicodemographic profile of kidney diseases were studied using statistical package for the social sciences version 20 and were represented as mean, standard deviation, number, percentage and ratio.

**Results:**

Out of a total of 829 patients, the commonest clinical syndrome and the histological patterns were end-stage renal disease 248 (29.9%) and IgA nephropathy 18 (20.7%) respectively. The mean age was 51.4±18.6 years. The commonest reason for hospitalization was sepsis 372 (44.8%). Males were 486 (58.6%) and females were 343 (41.4%).

**Conclusions:**

The commonest clinical presentation and the reason for admissions were end-stage renal disease and sepsis syndrome respectively.

## INTRODUCTION

The spectrum of kidney diseases includes various aspects of renal disorders and differs significantly in developing and developed countries. If left untreated they may lead to renal failure that may require renal replacement therapy, which is extremely expensive and places a severe burden on the healthcare system of the country.^1^

Kidney diseases have become a major public health problem globally and in Nepal too.^2–5^ and the estimated prevalence of chronic kidney disease (CKD) is around 10.6% but is expected to be higher.^4,5^ In a recent metaanalysis of AKI, the incidence of AKI was found to be 7.5% in Southern Asia and 31.0% in South-eastern Asia;^6,7^ However there are no such data from Nepal.

We had realized kidney diseases were a significant health problem in our region. However, there are no data regarding the nature of such patients in our center. We, therefore, thought of doing a study to know the clinicodemographic spectrum of kidney disease patients admitted to our hospital.

## METHODS

This study was a descriptive cross sectional study carried out in the department of Nephrology over one year, from May 2018 to April 2019. The ethical clearance for conducting the study was taken from the Institutional review committee of the hospital with the Ref no. 2016/ COMSTH/IRC/049. Convenient sampling was done and all the consecutive kidney disease patients, who were admitted in the department of Nephrology, irrespective of their age, sex, and renal diagnosis, were included in the study. The clinical diagnosis of renal diseases was made by a nephrologist with an experience of > 5 years in clinical Nephrology and all the diagnoses were supported by relevant biochemistry, radiology, and pathology reports. The prevalence of chronic kidney disease (CKD) is around (p) is 10.6%^4^, taking 95 % CI and 2.5% margin of error then sample size was calculated by using formula,

$$\begin{array}{ll} \text{n} & \text{= }{\text{Z}}^{\text{2}}\times \text{p}\times \text{q}/{\text{e}}^{\text{2}}\\ & \text{= }{\left(1.96\right)}^{\text{2}}\times (0.106)\times (1-0.106)/{(\text{0.025)}}^{\text{2}}\\ & \text{= }582 \end{array}$$

By taking 10% non-response error the actual sample size of this research was 642 but this research was conducted among 829 patients.

The standard definitions were used to define the renal diagnosis as per the updated kidney disease improving global outcome (KDIGO) equivalent criteria, wherever applicable. Written informed consent was taken from all the patients.. The patient's demographic profile, clinical diagnosis, comorbidities, the reason for hospital admissions, length of hospital stay, and the number of repeat admissions were noted in the proforma. The data were then entered in the MS XP sheet and were transferred to statistical package for social sciences version 20 (Chicago, IL, USA) program for analysis. The data were analyzed using mean, standard deviation, number, percentage and ratio.

## RESULTS

A total of 829 patients were admitted within a period of one year from April 2018 to May 2019. The commonest clinical syndrome and the histological patterns were end-stage renal disease 248 (29.9%) and IgA nephropathy 18 (20.7%) respectively. The commonest reason for hospitalization was sepsis 372 (44.8%). Out of them, males were 486 (58.6%) and the females were 343 (41.4%). The mean age of the patient was 51.4±18.6 years. The minimum age was 9 years and the maximum age was 93 years (Table 1). Of 829 patients, 246 (29.7%) were of age <40 years and 583 (70.3%) were of age ≥ 40 years. Further age distributions were as shown in the table below.

**Table 1:** Age distribution of the admitted patient (n = 829).

Age (in years)	Male n (%)	Female n (%)	Total n (%)
1-20	40 (68.9)	18 (31.1)	58 (6.9)
21-40	85 (45.2)	103 (54.8)	188 (22.7)
41-60	162 (55.1)	132 (44.9)	294 (35.5)
61-80	173 (67.8)	82 (32.2)	255 (30.7)
>80	26 (76.5)	8 (23.5)	34 (4.1)

In ESRD group (n = 248), the common causes were Type 2 diabetes mellitus (T2DM) 94 (37.9%), chronic glomerulonephritis (CGN) 75 (30.2%), hypertension (HTN) 63 (25.4%), autosomal dominant polycystic kidney disease (ADPCKD) 8 (3.2%), obstructive uropathy 7 (2.8%), and neurogenic bladder 1 (0.4%). Out of the 248 (29.9%) ESRD patients, 66 (26.7%) patients were diagnosed ESRD for the first time (i.e. new ESRD) and the remaining 182 (73.3%) were old ESRD, who were already on maintenance hemodialysis. A total of 313 (37.7%) patients received hemodialysis during the hospital stay, out of which 65 (7.8%) patients were in acute on CKD group and the remainders were ESKD. Among the new ESRD patients (n = 66) also, the common causes were T2DM 29 (43.9%) followed by CGN 28 (42.4%) and HTN 5 (7.5%) (Table 2).

**Table 2:** The clinical syndromes in the admitted patients (n = 829).

Clinical Syndromes	n (%)
End-stage kidney disease (ESRD)	248 (29.9)
Acute on CKD (Chronic kidney disease)	194 (23.4)
Acute kidney injury (AKI)	154 (18.6)
Chronic kidney disease (CKD)	85 (10.2)
Lupus nephritis (LN)	52 (6.3)
Nephrotic syndrome	13 (1.6)
Nephrotic/Nephritic syndrome	13 (1.6)
Rapidly progressing glomerulonephritis (RPGN)	12 (1.4)
Subnephrotic range proteinuria	5 (0.6)
Uncontrolled diabetes mellitus	5 (0.6)
Asymptomatic microscopic hematuria	4 (0.5)
Uncomplicated urinary tract infection	4 (0.5)
Wasp sting	3 (0.4)
Acute nephritic syndrome	2 (0.2)
Episodic gross hematuria	1 (0.1)
Henoch Schonlein purpura (HSP)	1 (0.1)
Uncontrolled hypertension	1 (0.1)

In acute on CKD (n = 194) group, the causes of acute kidney injury were complicated urinary tract infection 105 (54.1%), pneumonia 66 (34.0%), acute gastroenteritis (AGE) 13 (6.7%), cellulitis 5 (2.6%), tonsilopharyngitis 2 (1.0%), non-localizing sepsis 1 (0.5%), renal abscess 1 (0.5%), and melioidosis 1 (0.5%). The causes of CKD in acute on CKD group were Type 2 diabetes mellitus (T2 DM) 82 (42.2%), HTN51 (26.3%), obstructive uropathy 33 (17.0%), chronic glomerulonephritis (CGN) 23 (11.8%), neurogenic bladder4 (2.0%), and lupus nephritis 2 (1.0%).

In AKI group (n = 154), the causes of AKI were complicated UTI53 (34.4%), pneumonia 42 (27.3%), acute gastroenteritis (AGE) 18 (11.7%), non-localizing sepsis 9 (5.8%), scrub typhus 8 (5.2%), unexplained AKI requiring kidney biopsy 7 (4.5%), cellulitis 5 (3.2%), post-operative surgical site infection (SSI) 3 (1.9%), wasp sting 3 (1.9%), traumatic rhabdomyolysis 2 (1.3%), dengue 1 (0.64%), leptospirosis 1 (0.6%), drug induced acute interstitial nephritis 1 (0.6%), multiple myeloma 1 (0.6%). Of the 829 patients, the overall CKD patients were 527 (63.6%), (including the patients from ESKD, acute on CKD and CKD 3-5 group). The causes of CKD in this overall group (n= 527) were T2DM 208 (39.4%) followed by HTN 137 (25.9%) and CGN 123 (23.3%). Of the 829 patients, 87 (10.5%) patients had undergone kidney biopsy.

The indications of kidney biopsies were nephrotic syndrome 20 (22.9%), unexplained CKD 19 (21.8%), nephrotic/nephritic syndrome 13 (14.9%), lupus nephritis 12 (13.8%), unexplained AKI 7 (8.0%), rapidly progressing glomerulonephritis 5 (5.7%), asymptomatic microscopic hematuria 4 (4.6%), subnephrotic range proteinuria 4 (4.6%), nephrotic range proteinuria without other features of nephrotic syndrome 1 (1.1%), acute nephritic syndrome 1 (1.1%), and episodic macroscopic hematuria 1 (1.1%).

Most of the histological patterns of the kidney biopsies were of IgA nephropathy 18 (20.7%) followed by lupus nephritis 11 (12.6%), minimal change disease 9 (10.3%), membranous glomerulonephropathy 7 (8.0%) (Table 3).

**Table 3:** Histological patterns of kidney biopsies (n = 87).

Histological pattern of Kidney Biopsy	n (%)
IgA nephropathy	18 (20.7)
Lupus nephritis (LN)	11 (12.6)
Minimal change disease (MCD)	9 (10.3)
Membranous glomerulopathy (MGN)	7 (8.0)
Post Infectious diffuse proliferative glomerulonephritis	6 (6.9)
Focal Segmental glomerulosceloris (FSGS)	5 (5.7)
Hypertensive nephrosclerosis	5 (5.7)
Unexplained chronic glomerulonephritis (CGN)	4 (4.6)
Acute tubular necrosis (ATN)	4 (4.6)
Diabetic nephropathy	4 (4.6)
Thin basement membrane disease (TBMD)	4 (4.6)
Idiopathic cresentic glomerulonephritis	3 (3.4)
Myeloma kidney	3 (3.4)
C3 glomerulopathy	2 (2.9)
Chronic interstitial nephritis	1 (1.1)
Amyloidosis	1 (1.1)

The comorbidities associated were hypertension 452 (54.5%), benign enlargement of prostate (BEP) 49 (5.9%), hypothyroidism 47 (5.6%), chronic obstructive pulmonary disease (COPD) 40 (4.8%), Type 2 diabetes mellitus 28 (3.4%), ischemic heart disease (IHD) 25 (3.0%), carcinoma cervix 16 (1.9%), pulmonary tuberculosis 16 (1.9%) and rheumatoid arthritis 14 (1.7%).

Of the sepsis patient (n= 372), the causes of sepsis were complicated UTI 167 (44.9%), pneumonia 148 (39.8%), acute gastroenteritis 13 (3.5%), cellulitis 10 (2.7%), non-localizing sepsis 10 (2.7%), scrub typhus 8 (2.1%), catheter related blood stream infection 7 (1.9%), post-operative surgical site infection 3 (0.8%), tonsilopharyngitis 2 (0.5%), leptospirosis 1 (0.3%), dengue 1 (0.3%),renal abscess 1 (0.3%), and melioidosis 1 (0.3%) (Table 4).

**Table 4:** Common reasons for hospital admission of the patients (n = 829).

Reason of admission	n (%)
Sepsis	372 (44.8)
Volume overload alone	136 (16.4)
Volume overload with heart failure	55 (6.6)
Nephrotic syndrome evaluation	45 (5.4)
Uremic gastritis	27 (3.2)
Intra venous cyclophosphamide pu lse	26 (3.1)
CKD evaluation	26 (3.1)
Nephrotic/ nephritic syndrome evaluation	13 (1.6)
RPGN evaluation	12 (1.4)
Hypoglycemia	9 (1.0)
SLE flare	7 (0.8)
Gouty attack of joint	6 (0.7)
Subnephrotic range proteinuria evaluation	5 (0.6)
Uncontrolled DM	5 (0.6)
Asymptomatic microscopic hematuria evaluation	4(0.5)
Uncomplicated UTI	4(0.5)
Wasp sting	3 (0.4)
Intravenous rituximab therapy	3 (0.4)
Hyperkalaemia	2 (0.2)
Acute nephritic syndrome	2 (0.2)
Episodic gross hematuria	1 (0.1)
Henoch Schonlein purpura (HSP)	1 (0.1)
Uncontrolled HTN	1 (0.1)

Patients with less than 5 days of hospital stay were 504 (60.8%), with 5-10 days of hospital stay were 243 (29.2%), and with >10 days of hospital stay were 82 (9.9%). The longest period of hospital stay was 21 days and the shortest period was 1 day. The average duration of hospital stay was 5.5±3.6 days. The patients who had repeat admissions of ≥2 times were 192 (23.1%). The repeat admissions of 2 times were 101 (12.1%), 3 times were 58 (6.9%), 4 times were 23 (2.7%), 5 times were 6 (0.7%), 6 times were 3 (0.4%) and 7 times was 1 (0.1%). The maximum number of readmission of a patient was 7 times.

## DISCUSSION

In Nepal, there are limited numbers of nephrologists and only a few dedicated nephron centers to provide the nephrology service to kidney patients. Our study was the first of its kind from Chitwan to know the nature of kidney diseases prevailing in this region. Of the 829 patients, the majority of the patients were males 486 (58.3%), with the male to female ratio of 1.4. Similar observations of male preponderance were seen in other studies from India.^8,9^ This dominance of males over the female may reflect the socio-dynamic influence of our society, where a treatment privilege goes to males or it may be because the males were inherently predisposed to develop kidney diseases. This area of research needs multicentric genetic studies.

The three most common clinical syndromes observed in our study were ESRD 248 (29.9%), Acute on CKD 194 (23.4%), and AKI 154 (18.6%). In a similar South African study done by van Rensburg, the main clinical presentations documented were Chronic renal failure (CRF) 461 (37.9%); nephrotic syndrome 203 (16.7%); hypertension 161 (13.2%), and abnormal urinary findings 128 (10.5%),^10^ projecting CKD/ESRD to be the commonest clinical presentation in different parts of the world including ours. The high burden of ESRD could be explained by the silent and asymptomatic nature of the disease, lack of population awareness about the disease, poorly equipped health care system, and high cost of treatment.^1,11^ Sixty-six (7.9%) patients were diagnosed ESRD for the first time in our study, making the incident ESRD a significant problem in our center. Similar to our study Sakhuja, et al.^12^ also reported about two-thirds of their patients to be new ESRD at the time of the first consultation.

The overall CKD patients including the patients from ESRD group, acute on CKD group, and CKD3-5 group were 527 (63.6%), which projects CKD as the dominant clinical syndrome. The observation of CKD being the dominant clinical syndrome in our study might be explained by the global increase in the prevalence of diabetes and chronic glomerulonephritis. Similar observations of CKD being the dominant presentation were also seen in Indian studies from North India (PGI,^13^ AIIMS,^14^ and SGPGI).^15^

The commonest cause of ESRD in our study was T2DM 94 (37.9%) followed by CGN 75 (30.2%) and HTN 63 (25.4%) highlighting the fact that T2DM is the number one cause of ESRD in our region and also globally.^16,17^ However, there seems to a considerable heterogeneity in the causes of CKD/ESRD within and across the country. Few studies from Nepal and India, found CGN to be the commonest cause of CKD.ESRD.^11,18^ Similarly a study done by Khakurel, et al. revealed glomerulonephritis and hypertension to be the most common causes for end-stage renal failure^19^ whereas Chhetri, et al. showed hypertension, diabetes mellitus and glomerulonephritis to be the most common causes of End-stage renal disease (ESRD).^20^ This heterogeneity in the causes merits a larger and multicentric trial to understand the true cause of ESRD prevailing in our country.

The commonest cause of AKI in our study was a complicated urinary tract infection 53 (34.4%) followed by pneumonia 42 (27.3%) and acute gastroenteritis (AGE) 18 (11.7%). However, in one study done by Khakurel, et al. from Nepal, the common causes of acute renal failure documented were gastroenteritis and sepsis.^21,22^ This non-uniform pattern of causes of AKI in different centers of Nepal creates confusion and this situation is complicated more by the fact that we still don't have central AKI registry. This highlights the importance of conducting a larger multicentric study to better understand the true nature of AKI prevailing in our country. Patterns of AKI are also different across Asia. In India, patients who develop CA-AKI are more frequently affected by infectious diseases (47%), including malaria in 17%, infectious diarrhea in 19%, and sepsis in 11%. Other common causes of CA-AKI in India include obstetric complications, animal and plant toxins, and the use of natural medicines.^23^ AKI is quite different in developed countries where cardiovascular procedures and sepsis in elderly persons are common cause.^24^

The most common comorbidity seen in our study was hypertension 452 (54.5%) followed by BEP 49 (5.9%). Similar; observations were made by Fraser, et al. where HTN was also the commonest comorbidity 87.8%.^25^ HTN being the commonest comorbidity is logical and can be explained pathophysiologically because of renin angiotensin aldosterone system (RAAS) activation and fluid and sodium retention.

The commonest reason for hospital admission was sepsis 372 (44.8%). This high light the burden of sepsis in our region and kidney disease patients especially ESRD are 100-300 times increased risk of infection.^26^ The second most common reason for hospitalization was volume overload without heart failure 136 (16.4%). This might be because of the patient's poor fluid compliance and treatment adherence which had predisposed them to increased risk of volume overload. The most common cause of sepsis was complicated UTI 167 (44.9%), followed by pneumonia 148 (39.8%). One study done in India by Sharma, et al.^27^ urinary tract was found to be the commonest focus of infection in 40 (40%) patients followed by pulmonary 31 (31%) and gastrointestinal tract 9 (9%) suggesting UTI be an important cause of sepsis.

The average duration of hospital stay in our study was 5.5±3.5 days. In the study done by Kshirsagar, et al.^28^ the average length of stay for hemodialysis patients under the care of nephrologists was 6.3 days, compared with 8.1 days under the care of internists with nephrology consultation.

In our study 193 (23.2%) patients had single admission, 101 (12.2%) had 2 admissions, 58 (7%) had 3 admissions, 23 (2.8%) had 4 admissions and 10 (1.2%) had 5 admissions. However in the study done by Kshirsagar, et al.^28^ 73% of patients had a single admission, 21% had two admissions, and 2% (three patients) had four admissions. The maximum number of readmissions in our study might suggest a high burden of infection in our region as sepsis was the commonest cause of admission. There were few limitations in our study. First this was an observational study hence; all the inherent limitations of an observational study were there in the study. This was also a single centre study, so the results might not be generalizable to whole region and the country, highlighting the need for multicentric studies with a uniform protocol.

## CONCLUSIONS

This study has helped us understand the nature and spectrum of kidney diseases prevailing in our region and can help formulate appropriate programmes and plans to tackle the common prevailing problems. However we need a multicentric study to understand the true nature and prevalence of the kidney diseases in the country.
